# Evaluation of socio-demographic and clinical characteristics of PCOS patients attending a tertiary care institute in Colombo

**DOI:** 10.1186/s12902-022-01206-0

**Published:** 2022-11-21

**Authors:** I. Ranathunga, T. G. Athukorala, M. R. Sumanatilleke, N. P. Somasundaram

**Affiliations:** Diabetes and Endocrinology Unit, National Hospital of Sri Lanka, Colombo 10, Sri Lanka

**Keywords:** PCOS, Socio-demographic, Clinical, Hyperandrogenism, Irregular menstrual cycles, Tertiary care institute in Sri Lanka

## Abstract

**Background and objectives:**

Polycystic ovary syndrome (PCOS) is a common endocrine disorder with heterogeneous aetiology. It is characterized by irregular menses and or oligo/anovulation, hyper-androgenism, and polycystic ovaries. The prevalence and diagnosis of PCOS changes depending on which clinical criteria are utilized to confirm the diagnosis. The prevalence can be high as 8–13% when the Rotterdam criteria are used. However, there is significant inter-individual variation in presentation. We have studied the socio-demographic and clinical characteristics of PCOS patients attending the Endocrinology clinic in a tertiary care institute in Sri Lanka.

**Methods:**

A descriptive cross sectional study was conducted from September 2019 to September 2020 at the Endocrinology Unit of the National Hospital of Sri Lanka. All the patients who met the inclusion and exclusion criteria and who has a diagnosis of PCOS made according to Rotterdam criteria were recruited in to the study. After obtaining informed written consent, the data was collected using an interviewer administered questionnaire. HOMA-IR was calculated using the fasting insulin and blood glucose level.

**Results:**

The study enrolled sixty females. The mean age was 26.7 years (range 18–44). The mean weight was 64.8 (SD = 11.9) kg and BMI was 27.1 (SD = 4.8) kg/m^−2^. According to Asian BMI cut-offs, 1 (1.7%) patient was underweight and 13 (21.7%) had normal weight. Forty six (76.7%) had their weight in the overweight or obese category. Fifty four (90.0%) patients had clinical or biochemical evidence of hyperandrogenism while 24 (40%) had polycystic ovaries on trans-abdominal ultrasound scan and 50 (83.3%) had irregular menstrual cycles. According to the body fat percentage assessed by the whole body DEXA scan 4.1% normal body fat, while 50.0% and 45.8% had overweight and obesity respectively. HOMA-IR detected 61.1% to have high insulin resistance. Out of the patients who had USS of the abdomen 27.5% had co-existent non-alcoholic fatty liver. Fifty four percent of the patients had sub/infertility.

**Conclusions:**

The majority of the population were overweight or obese and had higher prevalence of insulin resistance and non-alcoholic fatty liver. Out of the clinical characteristics used to make the diagnosis of PCOS, the presence of clinical or biochemical evidence of hyperandrogenism and irregular menstrual cycles are more common than the detection of polycystic ovaries on trans-vaginal USS. The higher prevalence of overweight, obesity, insulin resistance and NAFLD associated with PCOS makes the diagnosis and management of the disease crucial to prevent long term consequences of the disease.

## Background

Polycystic ovary syndrome (PCOS) is considered as the most common endocrine disorder prevalent in females. Multiple genetic and environmental factors play a complex role in its aetiology. Patients can present with varied symptoms, but predominant clinical manifestations include irregular menses and or oligo/anovulation, hyper-androgenism, and polycystic ovaries. The prevalence of PCOS is variable in different populations of people with varied geography and ethnicity. Moreover, the different criteria that are in practise to diagnose the disease contribute to the differences in the prevalence among different groups of people that are observed. The widely used Rotterdam criteria diagnose the disease in about 8–13% of females [1]. In the western countries, polycystic ovarian syndrome (PCOS) has a prevalence of 4–12% rendering it as the most prevalent endocrine disorder of reproductive-age women [2]. A prevalence of 6.5–8% has been reported in european countries [3].

Clinical manifestations of the disease include menstrual irregularities, hirsutism, and commonly infertility or subfertility. Commonly seen menstrual irregularities in PCOS patients include prolonged erratic menstrual bleeding, amenorrhea and oligomenorrhea [4]. Nevertheless, some of the females with PCOS will have normal menstrual cycles with or without anovulation [5]. On presentation, majority of females with oligomenorrhea and around half of females with amenorrhea will have PCOS [6]. Majority of females, with clinical features of androgen excess will eventually be diagnosed to have PCOS [7]. Androgen excess can be characterized by hirsutism, acne, androgenic pattern of hair loss etc. Hirsutism, which is excessive growth of terminal hair, is a frequent clinical feature of hyperandrogenism that can be seen in majority of females with PCOS [8]. Hirsutism can be objectively assessed using a modified Ferriman–Gallwey scoring system [9]. Majority (over 90%) of females with normal menstrual cycles and hirsutism are identified through ultrasound to have polycystic ovaries [10]. Furthermore, PCOS can be detected in 50% of women without significant hirsutism [11]. Acne is also a clinical feature indicative of hyperandrogenism, but is uncommonly seen in PCOS. Around one third of females with PCOS will have acne on diagnosis [7, 12]. Around forty percent of females with severe acne will have the diagnosis of PCOS made on presentation [13]. Infertility can occurs in about half of females with PCOS [14]. Though patients diagnosed with PCOS have a normal number of primordial follicles, they have significantly increased number of their primary and secondary follicles. Arrest in follicular growth can be possibly described by alterations in the factors involved in normal follicular development such as premature luteinisation of granulosa cells and suboptimal follicular stimulating hormone (FSH) secretion in PCOS [15]. This will lead to formation of increased number of follicles with a diameter of 5–8 mm [15]. As a mature follicle does not form, successful ovulation with normal fertility does not occur [14]. Furthermore, increased incidence of spontaneous abortion is also seen in PCOS [16].

PCOS diagnostic criteria had been evolved over past few decades. Up to present, three sets of diagnosticcriteria are available for the diagnosis. The first criteria that had been described is the National Institutes of Health/National Institute of Child Health and Human Disease (NIH/NICHD) criteria followed by the European Society for Human Reproduction and Embryology/American Society for Reproductive Medicine (ESHRE/ASRM); Rotterdam criteria; and the latest Androgen Excess and PCOS Society diagnostic criteria [17–19]. All three diagnostic groups consider PCOS as a diagnosis of exclusion. Other alternative diagnoses such as Cushing syndrome, hyperprolactinemia, thyroid disorders congenital, adrenal hyperplasia, non-classic adrenal hyperplasia, androgen-secreting tumour, idiopathic hirsutism and idiopathic hyperandrogenism must be eliminated prior to the diagnosis of PCOS.

Pathogenesis of the development of PCOS is multifactorial. Family history has been described as a possible risk factor for PCOS based on the accumulation of cases within families [20]. Increased diagnosis of PCOS or its clinical features among first-degree relatives is indicative of genetic predisposition [21]. Furthermore, higher concordance has been detected in monozygotic versus dizygotic twins [22].

PCOS is connected with numerous other disease conditions. Fat accumulation with development of overweight and obesity often precedes the appearance of the clinical manifestations of PCOS. Adhering to a healthy lifestyle with dietary modifications and exercise therapy has been shown to decrease weight, improve insulin resistance, decrease abdominal fat, decrease testosterone and improve features of hyperandrogenism in females with PCOS [23, 24].Diabetes mellitus including Type 1, Type 2, and gestational diabetes is linked with a higher occurrence of PCOS. Codner et al. screened 42 women with type 1 diabetes mellitus for detection of PCOS using the Rotterdam criteria and compared with body mass index (BMI) matched controls [25]. The detection of PCOS was higher in the type 1 diabetes patients group compared to the control group, giving rise to a relative risk of PCOS of 15.4 (95% confidence interval [CI] 2.2–110.2; *P* < 0.0001) in the diabetic patients group. The prevalence of the disease in type 2 diabetes patients has been found to be 26.7% [26]. The presence of PCOS was 16% in patients with gestational diabetes (*P* = 0.03) [27]. PCOS patients with insulin resistance has an increased the risk of developing metabolic syndrome, reproductive dysfunction and epilepsy [28, 29]. A number of prenatal and postnatal factors have been connected with a higher likelihood of occurrence of PCOS in children [30]. Prenatal predispositions include increased birth weight, evidence of congenital virilization, and decreased birth weight [30]. Factors that may be evident in adolescence include premature pubarche, atypical central precocious puberty, obesity syndromes, acanthosis nigricans, and metabolic syndrome [30].

An increased risk of development of dyslipidemia has been demonstrated in PCOS. Lipid abnormalities that has been found include elevated triglycerides (TG), decreased high density lipoprotein-cholesterol (HDL-C) and elevated low density lipoprotein-cholesterol (LDL-C) [31]. Furthermore, numerous studies has shown that females with PCOS have a higher incidence of hypertension [32]. Morbidity and mortality from coronary heart disease among women with polycystic ovary syndrome is not as high as previously predicted. This finding challenges our understanding of the aetiology of coronary heart disease in women with the disease [33].

Though there are several international studies done on the prevalence, clinical features and associations of PCOS, the Sri Lankan data on the subject is still sparse. This study is done to evaluate the socio-demographic and clinical characteristics of the patients with PCOS attending a tertiary care institute in Sri Lanka. Furthermore, risk factors for disease development as well as associated comorbidities such as diabetes, dyslipidaemia, hypertension, obesity, metabolic syndrome, cardiovascular diseases, non- alcoholic fatty liver disease and mental health diseases are evaluated in this study.

## Methods

A descriptive cross sectional study was carried out by recruiting adult females aged > 18 years, who are pre-menopausal and diagnosed with PCOS and attending the Endocrinology clinic at National Hospital of Sri Lanka. The clinical assessments and tests were performed by trained researchers. The urban and suburban residents living in Colombo attend the above clinic and the study sample represent the above population.

### Sample size

As Endocrinology clinic of the National Hospital of Sri Lanka is a tertiary care centre as well as a referral centre, we have noted that on average about 6–10 patients with already diagnosed or newly diagnosed PCOS attend the clinic each month. As the data is being collected over 10 months starting from November/2019 to September/2020, sixty patients were enlisted to the study.

### Sampling technique

Females aged > 18 years and premenopausal, who has a previous or a new diagnosis of PCOS according to the Rotterdam criteria without prior treatment were recruited to the study [18].

According to the European Society for Human Reproduction and Embryology/American Society for Reproductive Medicine (ESHRE/ASRM)/ Rotterdam criteria/ 2003, fulfilment of two out of three of the below criteria is necessary to make the diagnosis of PCOS, after excluding the other conditions that mimic the disease such as Cushing syndrome, hyperprolactinemia, thyroid disorders congenital, adrenal hyperplasia, non-classic adrenal hyperplasia, androgen-secreting tumour, idiopathic hirsutism and idiopathic hyperandrogenism [18].

- Oligo- and/or anovulation
- Clinical and/or biochemical signs of hyperandrogenism
- Polycystic ovaries (by ultrasound)

Females who are pregnant and who has not given informed written consent were excluded from the study. All the participants meeting the above mentioned inclusion and exclusion criteria was included to the study over a period of ten months.

### Data collection

The selected participants who were attending the endocrine clinic at the National Hospital of Sri Lanka was educated regarding the study by a team of researchers to explain about the research and to invite them for the study. Informed written consent was taken by the interviewer and the data was collected with the aid of an interviewer administered questionnaire administered by trained researchers and by measurement of necessary anthropometric measures (weight, height, waist circumference, modified Ferriman Gallway score, blood pressure). Body Mass Index (BMI) was calculated by dividing weight in kilograms by height in meters squared (kg/m2). All the participants were evaluated with Luteinizing hormone (LH) (Chemiluminescent immunoassay analyser), Follicular stimulating hormone (FSH) (Chemiluminescent immunoassay analyser), serum total Testosterone (Fully automated immunoassay analyser ADVIA centaur XP), fasting plasma glucose (FPG) (GOD- PAP5 method, Abbott architect analyser), Post prandial blood glucose (PPBG) (GOD- PAP5 method, Abbott architect analyser), Glycated haemoglobin levels (HbA1c) ( (HPLC method, HPLC analyser), Fasting Lipid profile including total cholesterol (TC), Triglyceride (TG) and Low density lipoprotein- cholesterol (LDL-C) (Fully automated chemistry analyser ABBOTT architect plus C8000), Aspartate aminotransferase (AST) (Fully automated chemistry analyser Bechman Coulter- AU 680), Alanine aminotransferase (ALT) (Fully automated chemistry analyser Bechman Coulter- AU 680) and serum fasting insulin (Chemiluminescent enzyme immunoassay, Immulite 1000 analyser) measurements. Homeostatic Model Assessment of Insulin Resistance (HOMA-IR) was calculated using the fasting insulin and blood glucose level [34]. Body fat percentage was measured using the whole body Dual-energy x-ray absorptiometry (DEXA) scan.

### Statistical analysis

Data was analysed using Statistical Package for Social Sciences 18. Descriptive data was used to describe the population characteristics. Chi-square test was used to compare categorical variables.

### Ethical issues

Ethical approval was obtained from the ethical review committee of the National Hospital of Sri Lanka as well as by the ethics review committee of the post graduate institute of medicine, University of Colombo, Sri Lanka (ERC/PGIM/2020/029). Documents were encoded with numerical values to avoid personal identification. All the measures were taken to ensure confidentiality of subjects.

### Definitions

Modified Ferriman Gallway scores more than 8 is considered abnormal [9]. Furthermore, scores between 8–15 are considered as mild hirsutism, 16–25 as moderate, and scores > 25 as severe hirsutism [9]. The diagnosis of diabetes was made if FPG ≥ 126 mg/dL (7.0 mmol/L), or 2-h plasma glucose ≥ 200 mg/dL (11.1 mmol/L) during an OGTT or HbA1C ≥ 6.5% according to the American Diabetes Association 2020 criteria [35]. In the age group of 20–39 years of Asian females, body fat > 35% is considered as overweight and > 40% is considered as obese while in the age group of 40- 59 years of Asian females, body fat > 36% is considered as overweight and > 41% is considered as obese [36].

## Results

60 participants who met the inclusion and exclusion criteria was included in to the study. Mean age of the population was 26.7 (SD ± 6.7) years with a minimum age of 18 years and the maximum age of was 44 years. The socio-demographic characteristics are summarised in Table 1.

**Table 1:** Study group Socio-demographic characteristics

		Number (*N* = 60) (%)
Ethnicity % (N)	Sinhalese	47 (78.3)
	Muslim	8 (13.3)
	Sri Lankan Tamil	2 (3.3)
	Others	3 (5.0)
Education level % (N)	No education	1 (1.7)
	Grade 5	2 (3.3)
	Ordinary Level	21 (35.0)
	Advanced Level	24 (40.0)
	Tertiary	12 (20.0)
Employment status % (N)	Employed	29 (48.3)
	Unemployed	31 (51.7)
Marital status	Married	20 (33.3)
	Unmarried	39 (65.0)
	Other	1 (1.7)

The mean weight is 64.8 (SD ± 11.9) kg and BMI is 27.1 (SD ± 4.8) kg/m^−2^. The mean waist circumference is 64.9 (SD ± 28.8) cm. The study group anthropometric measurements are summarised in Table 2.

**Table 2:** Study group Anthropometric measurements

	Minimum	Maximum	Mean	Standard Deviation
Height (cm)	125	172	155.2	7.5
Weight (kg)	40	103	64.8	11.9
Waist circumference (cm)	28	105	64.9	28.8
BMI (kg/m2)	17.2	41.8	27.1	4.8

**Fig. 1:**
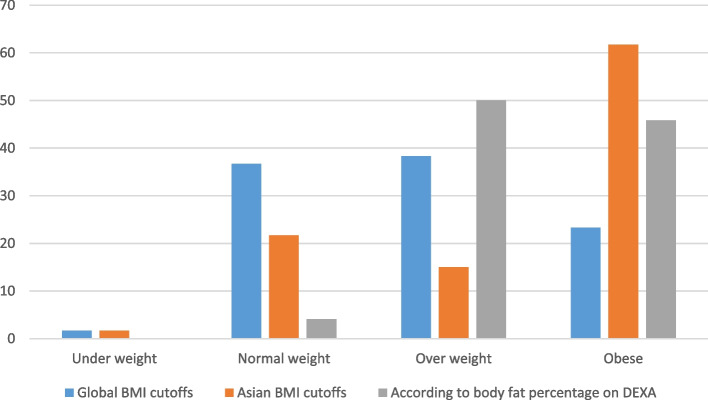
Prevalence (%) of underweight, normal weight, overweight and obesity according to Asian, Global BMI cut-offs and body fat percentage on DEXA scan

According to Asian BMI cut-offs, 1 (1.7%) patient was underweight and 13 (21.7%) had normal weight [37]. Forty six (76.7%) had their weight in the overweight or obese category. Body fat percentage measurement on DEXA scan detected 50.0% as overweight and 45.8% as obese (Fig. 1) [36].

The study extensively looked at the group characteristics such as birth weight, pubarche and menarche of the study participants. Hirsutism was assessed using the modified Ferriman Gallway scoring system. All the patients underwent multiple laboratory tests including LH, FSH, Serum total Testosterone, FBG, PPBG, Glycated haemoglobin (HbA1c), TC, TG, LDL-C, AST, ALT and Serum fasting insulin level as well as the body fat percentage by DEXA scan. The testosterone reference range for adults female is taken as 14–76 ng/dL according to the lab reference range. The results are summarized in the Table 3.

**Table 3:** Study group characteristics

	Minimum	Maximum	Mean	Standard. Deviation
Age	18	44	26.7	6.7
Birth Weight (Kg)	1.5	5.1	2.8	0.65
Pubarche age	9	18	11.9	1.7
Menarche age	9	18	12.2	1.7
Hirsutism (Ferriman Gallway Score)	3	26	14.5	5.5
LH Level (IU/L)	1.1	23.1	10.2	5.8
FSH Level (IU/L)	1.1	16.9	5.9	2.5
Serum Total Testosterone (ng/dL)	8.6	193	63.8	40.9
FBS (mg/dL)	66	345	96.8	42.9
PPBS (mg/dL)	77.8	172	103.8	27.2
HbA1C (mg/dL)	4.5	10.5	5.7	1.2
TC (mg/dL)	130	323	209.8	50.5
TG (mg/dL)	50	366	132.5	67.3
LDL-C (mg/dL)	62	141	133.7	42.5
AST (U/L)	11	214	35.5	37.2
ALT (U/L)	10	148	38.2	31.8
Serum fasting insulin (mIU/ml)	3.8	44.9	14.6	10.0
Fat % on whole body DEXA	29.6	47.9	39.0	4.6

Lab Reference ranges

LH (Adult females)- Follicular phase 1.9–12.5 IU/L, Midcycle phase 8.7- 76.3 IU/L, Luteal phase 0.5- 16.9 IU/L, Post menopausal 15.9–54 IU/L

FSH (Adult females)- Follicular phase 2.5- 10.2 IU/L, Midcycle phase 3.4 -33.4 IU/L, Luteal phase 1.5–9.1 IU/L, Post menopausal 23–116.3 IU/L

Total Testosterone 14–76 ng/dL

FBS 70–110 mg/dL, PPBS < 140 mg/dL, HbA1c ≥ 6.5%- Diabetes, 5.7%-6.4%- Prediabetes, < 5.7%- Normal, TC < 200 mg/dL, TG < 150 mg/dL, LDL < 100 mg/dL, AST < 35 U/L, ALT < 35 U/L, Serum fasting insulin 2–20 mIU/ml

Known associations of the development of the PCOS is summarised in the Table 4. 21.7% patients had a family history of PCOS while none had a history of epilepsy needing anti-epileptic medications of maternal anti mullerian hormone (AMH) administration in the second trimester.

**Table 4:** Known association of PCOS

Category	Number	Percentage (3.1%)
Family history of PCOS	13	21.7
Epilepsy	0	0.0
Maternal AMH administration in the 2nd trimester	0	0.0
Congenital adrenal hyperplasia (CAH)	1	1.7

Fifty four (90.0%) patients had clinical or biochemical evidence of hyperandrogenism while 24 (47%) had polycystic ovaries on trans-vaginal ultrasound scan and 50 (83.3%) had irregular menstrual cycles (Table 5). According to the Rotterdam criteria the presence of ≥ 12 follicles in each of two ovaries measuring 2–9 mm in diameter and/or ovarian volume ≥ 10 mL on trans-vaginal ultrasound scan (USS) is considered as polycystic ovaries [18]. Elevated LH: FSH ratio > 2 was seen in 20 (33.3%) patients although it is not a component of the diagnostic criteria.

**Table 5:** Diagnostic criteria for PCOS

Criteria		Number (%)	Number (%)
Clinical or biochemical hyperandrogenism	Hirsutism (Ferriman Gallway score ≥ 8)	53 (88.3%)	54 (90%)
	Elevated Serum Testosterone (≥ 76 ng/dL)	16 (26.6%)	
Polcystic ovaries on USS			24 (47%)
Menstrual irregularity			50 (83.3%)

**Fig. 2:**
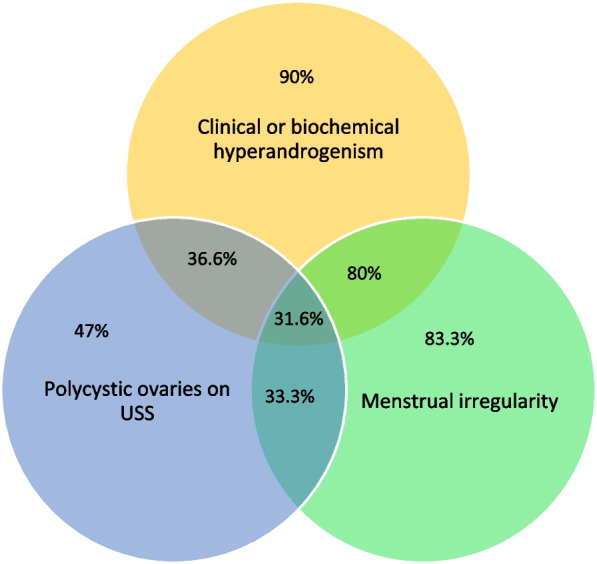
Overlap of the Rotterdam criterion used to diagnose PCOS

Clinical or biochemical hyperandrogenism and polycystic ovaries on USS was seen in 36.6% of the study group while 80% had clinical or biochemical hyperandroensim and menstrual irregularities. Menstrual irregularities and polycystic ovaries on USS was seen on 33.3% of the sample population. According to this findings it is evident that majority of the study population was diagnosed with PCOS using the presence of clinical and biochemical hyperandrogenism and menstrual irregularities. Acne is considered as a feature of hyperandrogenism and it was detected in 16 patients (26.7%). The results are summarised in the Fig. 2.

Out of the population, 4 patients (6.7%) was diagnosed to have diabetes mellitus though 14 patients (23.3%) had evidence of clinical insulin resistance demonstrated by the presence of Acanthosis nigricans. Few patients had pregnancy complications while 21.7% had subfertility/infertility. From the females who has actively tried to conceive, fifty four percent had sub/infertility. Out of the patients who had USS of the abdomen 27.5% (*n* = 14) had co-existent non-alcoholic fatty liver (Table 6).

**Table 6:** Known complications of PCOS

**Category**		**Number (*N* = 60)**	**Percentage from the whole population (%)**
Diabetes mellitus		4	6.7
Dyslipidemia		4	6.7
Hypertension		3	5.0
Cardiovascular disease		0	0.0
Cerebrovascular Disease		0	0.0
Venous thromboembolism		0	0.0
Mental Health disorders		0	0.0
Thyroid disease	Hypothyroidism	10	16.6
	Hyperthyroidism	3	5.0
Endometrial cancer		0	0.0
Pregnancy complications	Miscarriage	2	3.3
	Preterm birth	1	1.7
	GDM	1	1.7
	PIH	0	0.0
Non alcoholic fatty liver disease		4	6.7
Fertility	Normal	11	18.3
	Sub-fertile	12	20.0
	Infertile	1	1.7
Acanthosis nigricans		28	46.6
Fatty liver on USS		14	23.3

HOMA-IR detected 61.1% to have high insulin resistance. HOMA- IR value < 2 is considered as normal in the calculations [34].

When the above factors were compared according to the Asian BMI cutoffs, the presence of Acanthosis nigricans (*p* = 0.006), fatty liver on ultra sound scan (*p* = 0.005) had a significant association with the BMI category (Table 7). Furthermore, the presence of Acanthosis nigrians were significantly higher in clinically hirsute patients (*p* = 0.008).

**Table 7:** The relationship between the Asian BMI cut-offs and the known complications of PCOS

Asian BMI cut-offs	Underweight (n)	Normal weight (n)	Over weight (n)	Obese (n)	Significance (*p* value)
Complications of PCOS					
Acanthosis nigricans					0.006
Yes	0	1	4	23	
No	1	12	5	14	
Fatty liver on USS					0.005
Yes	0	0	1	13	
No	1	13	8	15	

## Discussion

PCOS remains as a significant health burden due to the associated metabolic, psychological and reproductive complications. The current study has demonstrated that 76.7% of the study group had their weight in the overweight or obese category according to the Asian BMI cut-offs [37]. Body fat percentage measurement on DEXA scan detected 50.0% as overweight and 45.8% as obese depicting the metabolic complications posed by the PCOS as well the future risk of development of an array of non –communicable diseases. In a study done by Hestiantoro et al. body fat percentage is found to be better marker for measuring inflammation in PCOS patients when compared to the BMI [38].

In a recent systemic review, a significantly higher proportion of dyslipidaemia, cardiovascular risk markers and metabolic syndrome was seen in patients with PCOS with higher BMI [39]. Nevertheless, the study populations has a low prevalence of chronic diseases such as diabetes, dyslipidaemia, hypertension, cardiovascular disease and cerebrovascular disease which can be explained by a relatively young age of the sample with a mean age of 26.7 (SD ± 6.7) and such analysis was not carried out. Majority of the study population has been educated up to or more than ordinary level indicating that the disease detection is higher in well-educated populations. In a study done in India the majority of PCOS fell in to the category of middle socio economic class [40]. Furthermore the majority (65%) were unmarried at the time of study which might indicate delays in marriage in these patients. Moreover, the study group had a mean FBG of 96.8 mg/dL (SD ± 42.9) and a HbA1c 5.7 (SD ± 1.2) which indicates the tendency towards development of frank diabetes. Furthermore, the diagnosis of PCOS increases insulin resistance contributed by the obesity associated with the disease [28]. The study group had a high prevalence of 61.1% of increased insulin resistance detected by the HOMA-IR assessment. Thus, intervening early to modify the risk factors can improve the future prospects of this relatively young group of individuals.

The commonest presentation of the disease is clinical or biochemical hyperandrogenism (90%) and menstrual irregularities (83.3%) in the study population as similar to the previous studies [4, 8]. Eighty percent of patients had a combination of above symptoms. Previous literature specifies acne as an uncommon clinical feature and was seen only in one third of the patients diagnosed with the disease [7]. The current study has demonstrated similar findings with only 26.7% patients suffering from acne. Nevertheless, high suspicion of the disease by the clinicians are paramount important as these are common clinical presentations of many other diseases.

The study has looked extensively in to the clinical, biochemical and radiological derangements that is associated with the disease. Although only 4 patients (6.7%) patients recruited to the study came up with a previously made diagnosis of dyslipidaemia, the study detected 18 patients (30.0%) to have a TC > 200 mg/dL, while 26 patients (43.3%) had LDL > 100 mg/dL indicting a higher prevalence of dyslipidaemia than previously diagnosed. The mean TC of the population is 209.8 mg/dL (SD ± 50.5) and the mean LDL is 132.5 mg/dL (SD ± 67.3) which is higher than the lab reference range. Furthermore, at the data collection, only 4 patients (6.7%) came up with a diagnosis of non-alcoholic fatty liver disease (NAFLD), while the study diagnosed NAFLD in 14 patients (23.3%). This corroborate the importance of detailed assessment of the patients diagnosed with PCOS in order to detect undiagnosed associated non communicable disease which might have disastrous complications in the future if not addressed early.

Reproductive complications associated with PCOS leads to numerous other problems including adverse psychological outcomes. The current study detected few patients to have had pregnancy complications while many more had concerns with subfertility/infertility (21.7%) consistent with the pathophysiology of the disease [14]. From the females who had or having fertility wishes fifty four percent had sub/infertility indicating the importance of addressing the fertility issues as well as the psychological effects arising from such complications of the disease.

The current study detected a significant association between high BMI categories and the presence of Acanthosis nigrians which is an indicator of insulin resistance and the presence of fatty liver on ultra sound scanning similar to the findings obtained in the recent systemic review [39]. Thus, addressing overweight and obesity might in turn will improve the detrimental metabolic parameters which will improve the long term outcome of PCOS patients.

The current study has several limitations. As this is a single centre study, the population may not represent the general population in the country which involve urban, sub-urban and rural areas. Furthermore, even though the study has extensively looked at clinical, biochemical, radiological features of the disease, we have not assessed the psychological impact of the diseases to the study participants. Moreover, as the study is a cross sectional study, follow up for the participant was not carried out to assess the response to the lifestyle and medical interventions that patients has undergone as a part of the routine management of the disease.

## Conclusions

The study detected the majority of the study population to be overweight and obese according to the BMI cut-offs as well as body fat percentage detected by whole body DEXA scan which indicates an alarmingly high incidence of metabolic complications occurrence in PCOS patients in the future. The most common manifestations of the disease includes the presence of clinical or biochemical evidence of hyperandrogenism and irregular menstrual cycles in contrast to the detection of polycystic ovaries on trans- vaginal USS which indicates the importance of clinical suspicion and clinical diagnosis of the disease. The higher BMI is associated with the presence of acanthosis nigricans and fatty liver which mark the importance of weight reduction in bringing down the insulin resistance and the development of fatty liver in patients with PCOS. The higher prevalence of non-communicable diseases in this group of young females marks the importance of the early diagnosis and management of not only the PCOS itself but also the associated metabolic, reproductive and psychological complications of the primary disease. Successful management of the disease will eventually ease the future health expenditure strain of the country. Future studies including multi-centre, prospective studies will contribute to the understanding of the clinical features and productive management of the disease.

## Data Availability

The data analyzed in this paper can be made available to researchers. Requests for access to the dataset used in this paper should be directed to the corresponding author.

## Abbreviations

PCOS: Polycystic ovary syndrome
NIH/NICHD: National Institutes of Health/National Institute of Child Health and Human Disease
ESHRE/ASRM: European Society for Human Reproduction and Embryology/American Society for Reproductive Medicine (ESHRE/ASRM)
BMI: Body mass index
FPG: Fasting plasma glucose
PPBG: Post prandial blood glucose
OGTT: Oral glucose tolerance test
HbA1C: Glycated haemoglobin
TG: Triglycerides
HDL-C: High density lipoprotein-cholesterol
LDL-C: Low density lipoprotein-cholesterol
LH: Luteinizing hormone
FSH: Follicular stimulating hormone
AST: Aspartate aminotransferase
ALT: Alanine aminotransferase
HOMA-IR: Homeostatic Model Assessment of Insulin Resistance
DEXA: Dual-energy x-ray absorptiometry
AMH: Anti mullerian hormone
CAH: Congenital adrenal hyperplasia
USS: Ultrasound scan
NAFLD: Non-alcoholic fatty liver disease
